# Machine Learning-Based Prognostic Signature in Breast Cancer: Regulatory T Cells, Stemness, and Deep Learning for Synergistic Drug Discovery

**DOI:** 10.3390/ijms26146995

**Published:** 2025-07-21

**Authors:** Samina Gul, Jianyu Pang, Yongzhi Chen, Qi Qi, Yuheng Tang, Yingjie Sun, Hui Wang, Wenru Tang, Xuhong Zhou

**Affiliations:** 1 Laboratory of Molecular Genetics of Aging & Tumor, Medical School, Kunming University of Science and Technology, 727 Jingming South Road, Kunming 650500, China; saminagul@kust.edu.cn (S.G.); jianyu_0898@163.com (J.P.); cyz1206414925@163.com (Y.C.); qiqi12047@163.com (Q.Q.); t1023063288@163.com (Y.T.); sunyj6039@163.com (Y.S.); huiwang266@163.com (H.W.); 2 State Key Laboratory of Primate Biomedical Research, Institute of Primate Translational Medicine, Kunming University of Science and Technology, Kunming 650500, China; 3 Office of Science and Technology, Yunnan University of Chinese Medicine, Kunming 650500, China

**Keywords:** breast cancer, stemness, regulatory T cells, machine learning, deep learning

## Abstract

Regulatory T cells (Tregs) have multiple roles in the tumor microenvironment (TME), which maintain a balance between autoimmunity and immunosuppression. This research aimed to investigate the interaction between cancer stemness and Regulatory T cells (Tregs) in the breast cancer tumor immune microenvironment. Breast cancer stemness was calculated using one-class logistic regression. Twelve main cell clusters were identified, and the subsequent three subsets of Regulatory T cells with different differentiation states were identified as being closely related to immune regulation and metabolic pathways. A prognostic risk model including *MEA1*, *MTFP1*, *PASK*, *PSENEN*, *PSME2*, *RCC2*, and *SH2D2A* was generated through the intersection between Regulatory T cell differentiation-related genes and stemness-related genes using LASSO and univariate Cox regression. The patient’s total survival times were predicted and validated with AUC of 0.96 and 0.831 in both training and validation sets, respectively; the immunotherapeutic predication efficacy of prognostic signature was confirmed in four ICI RNA-Seq cohorts. Seven drugs, including Ethinyl Estradiol, Epigallocatechin gallate, Cyclosporine, Gentamicin, Doxorubicin, Ivermectin, and Dronabinol for prognostic signature, were screened through molecular docking and found a synergistic effect among drugs with deep learning. Our prognostic signature potentially paves the way for overcoming immune resistance, and blocking the interaction between cancer stemness and Tregs may be a new approach in the treatment of breast cancer.

## 1. Introduction

Breast cancer ranks as the second most common disease on a global scale, according to the World Health Organization (WHO) reports [1]. Every year, women are diagnosed with approximately 268,600 new cases of invasive breast cancer, and about 41,760 women will die from breast cancer, as estimated by the American Cancer Society. Breast cancer is the sixth leading cause of cancer-related deaths among Chinese women, and approximately 11% of all breast cancers worldwide occur in China [1,2,3].

Regulatory T cells (Tregs) are a subset of lymphocytes characterized by the expression of the transcription factor FoxP3, playing a pivotal role in maintaining autoimmune tolerance, exhibiting strong immunosuppressive activity, as well as facilitating the initiation and progression of tumors by suppressing their antitumor immunity [4]. Tregs in the tumor microenvironment can inhibit dendritic cells, B cells, monocytes/macrophages, and other immunocompetent cells through cytotoxicity and cytokine secretion [5,6]. Tregs have been proven to promote the stemness of tumor cells, including those in glioma [7], leukemia [8], and breast cancer [9]. Cancer stem cells (CSCs) are a robust, heterogeneous population and the cellular sources of unlimited growth and recurrence of malignant tumors. Stemness plays a critical role in breast cancer growth, metastasis, and drug resistance [1,10]. The messenger RNA (mRNA) expression-based stemness index (mRNAsi) is used to quantify the unique characteristics of CSCs; Malta et al. developed a scoring system using one-class logistic regression (OCLR) machine learning algorithm as a robust method to quantify the cancer stemness [1,11,12,13]. In the new era of cancer treatment immunotherapies, immune checkpoint inhibitors (ICIs) have provided a paradigm shift and most immune checkpoint molecules are expressed in Tregs [14,15,16,17]. The effect of ICIs on Tregs and their contribution to treatment response remain unclear in breast cancer. We can accurately characterize breast cancer stemness and Regulatory T cells and identify the prognostic risk signature’s genes at a single-cell resolution to investigate better the impact of stemness and Tregs on ICIs. Here in this work, we established the prognostic risk model for breast cancer (BC). Our findings uncovered the potential of a prognostic risk model for predicting the prognosis, ICI outcomes, and immunotherapy effects of BC patients. Finally, the expression of genes in the prognostic risk model was validated using the HPA (Human Protein Atlas) database, and molecular docking and deep learning research were conducted to understand the performance of these genes and ways to improve them.

## 2. Results

### 2.1. Identification of Cell Types

**Figure 1:**
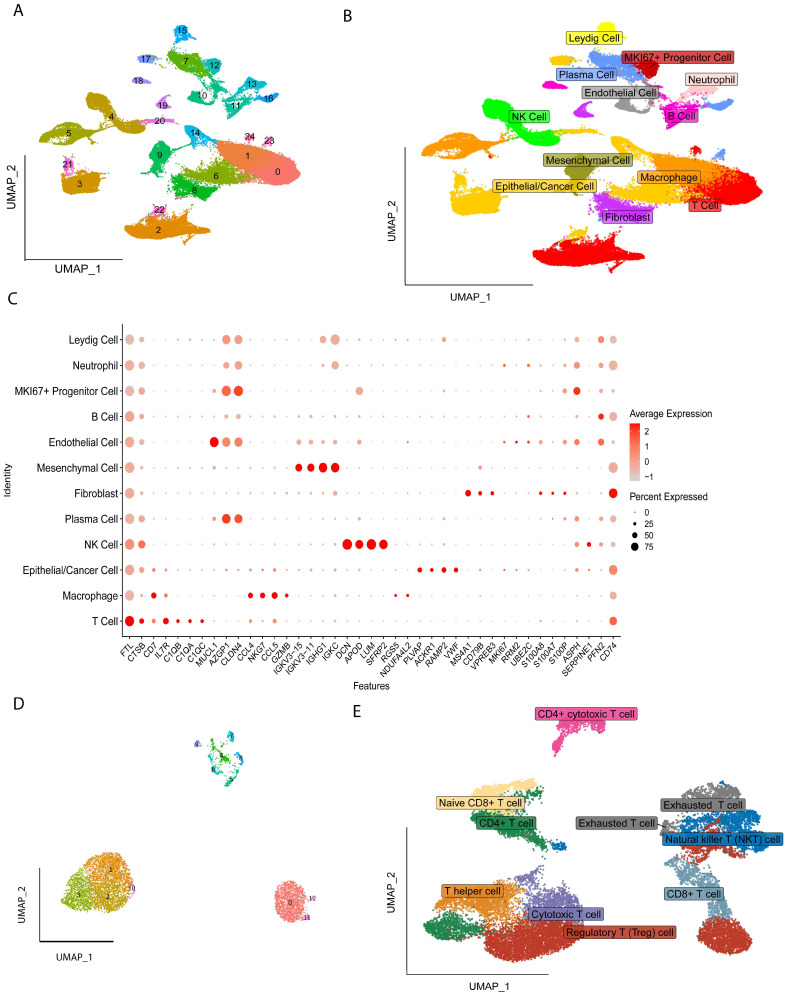
Single-cell analysis: Cell clusters and their markers were obtained by reduced-dimensional and clustering. (**A**) A total of 25 cell clusters were obtained after first-level classification. (**B**) A total of 12 cell types were obtained by marker gene annotation. (**C**) Expression levels of marker genes from 25 cell types. (**D**) A total of 13 cell clusters were identified after the second-level classification of T Regulatory cells. (**E**) Nine cell types were determined by marker gene annotation.

All the cells were clustered into 25 clusters by standard procedure (Figure 1A) and further annotated into twelve cell types, including Cancer/Epithelial cells, Macrophages, T cells, Mesenchymal cells, Endothelial cells, B cells, Neutrophils, Natural Killer cells (NK), Fibroblast, Leydig cells, MKI67+Progenitor cells, and Progenitor cells (Figure 1B), and the marker gene expression levels of the twelve cells are shown in Figure 1C, followed by further subgroup clustering of T Regulatory cells into thirteen clusters (Figure 1D), with nine additional subgroups identified by annotation: Regulatory T cells, Naïve CD8+ T cell, CD4+ T cell, T helper cell, Cytotoxic T cell, Exhausted T cell, Natural Killer T cell (NKT), CD4+ cytotoxic T cells and CD8+ T cell (Figure 1E).

### 2.2. Different Differentiation Characteristics of Tregs

**Figure 2:**
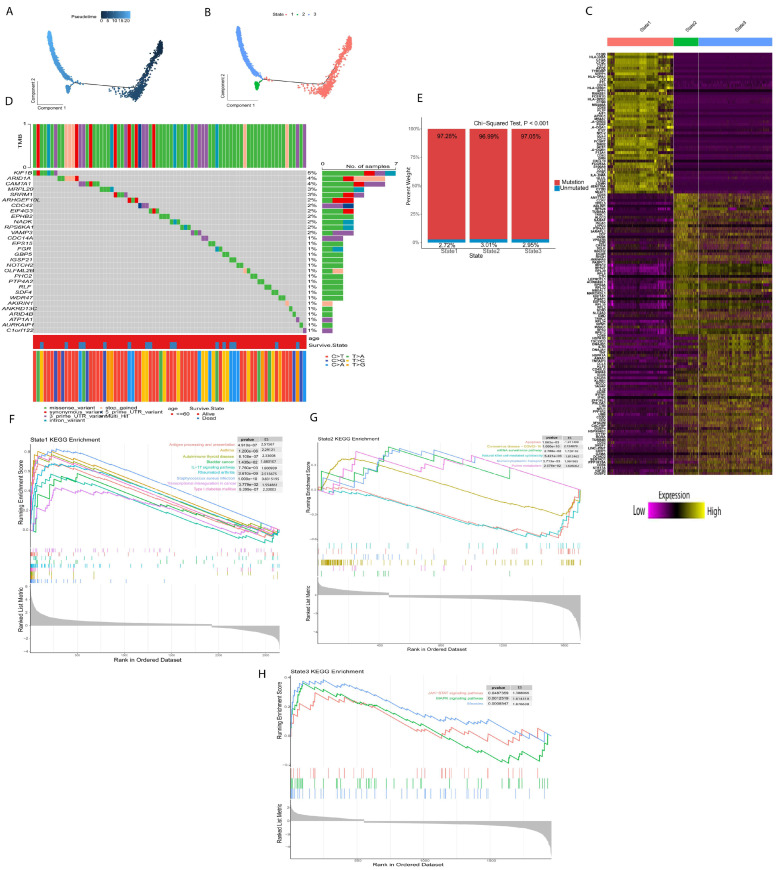
Mutational analysis of T regulatory cell-related differential genes and pseudotime analysis of Tregs. (**A**) According to pseudotime analysis (**B**), Tregs were divided into three differentiation states. (**C**) Treg-related differential genes in three differentiation states. (**D**) Top 30% mutation frequency of Treg-related differential genes. (**E**) Mutation of Treg-related differential genes in three differentiation states. (**F**–**H**) GSEA analyzes the three differentiation states of Tregs.

Next, we used Monocle for pseudotime trajectory analysis of Treg subsets; the results showed that Tregs were divided into three differentiation states (Figure 2A,B). The TRDGs related to the three states of Tregs are shown in (Figure 2C). The mutation rates of TRDGs in the three states were approximately 97%. The top 30 TDRGs of the twelve genes with a mutation rate ≥ 2% (Figure 2D,E) demonstrate that TDRGs are highly mutated and heterogeneous, suggesting that TDRGs play an essential role in Tregs influencing tumor growth and proliferation. The GSEA was performed on three states of Tregs (Figure 2F–H and Figure S1A–C); it was found that state one was significantly upregulated in autoimmune thyroid disease, bladder cancer, asthma, rheumatoid arthritis, and transcriptional mis-regulation in cancer, indicating that state one is mainly related to the development and occurrence of different disease and tumors. State two was upregulated in purine metabolism, mRNA surveillance pathways, and Coronavirus disease and downregulated in apoptosis and Natural killer cell-mediated cytotoxicity. State three is regulated in measles, JAK-STAT signaling pathway, and MAPK signaling pathways, indicating that state three is also related to the growth and development of tumors.

### 2.3. Development and Verification of Prognostic Risk Model

**Figure 3:**
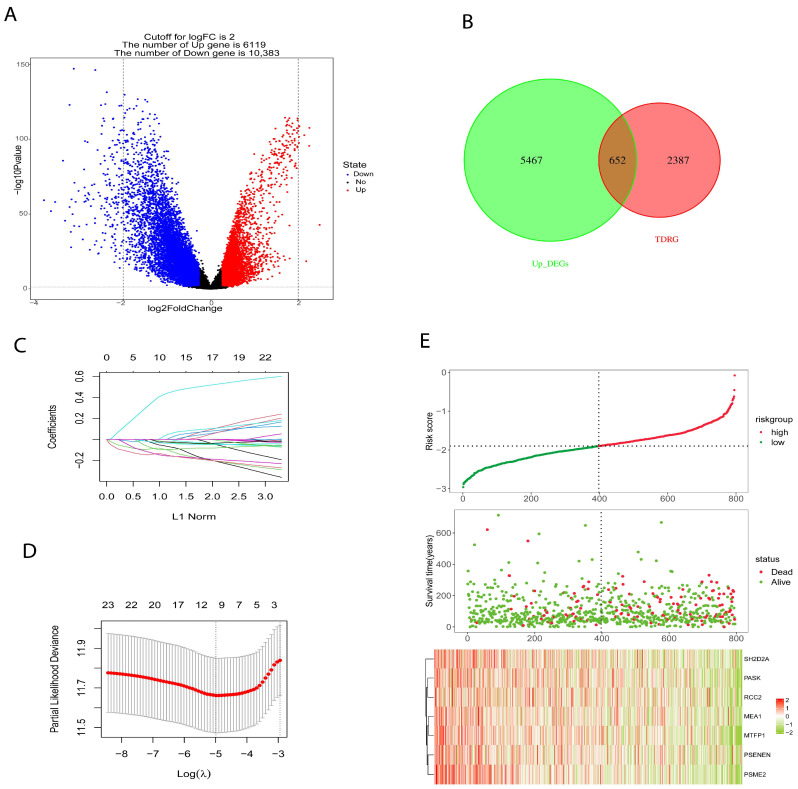
Construction and verification of prognostic risk signature model. (**A**) Differential genes between high- and low-mRNAsi (stemness index). (**B**) The intersection between differential genes related to stemness index and Treg-related differential genes. (**C**,**D**) Seven TDRGs (T regulatory cell differentiation-related genes) with prognostic characteristics were screened by the LASSO regression algorithm. (**E**) The risk score, patient status, and mRNA expression heatmap.

We used OCLR algorithm, calculated the stemness index (mRNAsi) for each patient in the BRCA cohort using RNA-seq data, and based on median value, patients were placed into high- and low-mRNAsi groups. “LIMMA” package was utilized to identify differential genes (DEGs) between high- and low-mRNAsi groups (Figure 3A) to construct a prognostic risk model. The TCGA cohort consist of 1217 samples (n = 1217) was split into training set (n = 796) and validation set (n = 421) at an equal ratio of 7:3. In addition, TDGRs and stemness-related DEGs were intersected (Figure 3B) and the intersected genes were used to build a prognostic risk model. Firstly, we identified 26 genes using univariate Cox regression analysis and reduced the number to 7 genes using LASSO regression (Figure 3C,D); then, we used these 7 genes to build a prognosis signature. The prognostic signature formula was as follows: Risk score = −0.049 × Exp (*MEA1*) − 0.082 × Exp (*MTFP1*) − 0.143 × Exp (*PASK*) − 0.0214 × Exp (*PSENEN*) − 0.132 × Exp (*PSME2*) − 0.096 × Exp (*RCC2*) − 0.030 × Exp (*SH2D2A*). According to the Risk score formula, we counted the risk score of all patients and assigned them to high-risk (n = 398) and low-risk (n = 398) groups using the median risk score. The association between survival and risk score information is exhibited in Figure 3E.

**Figure 4:**
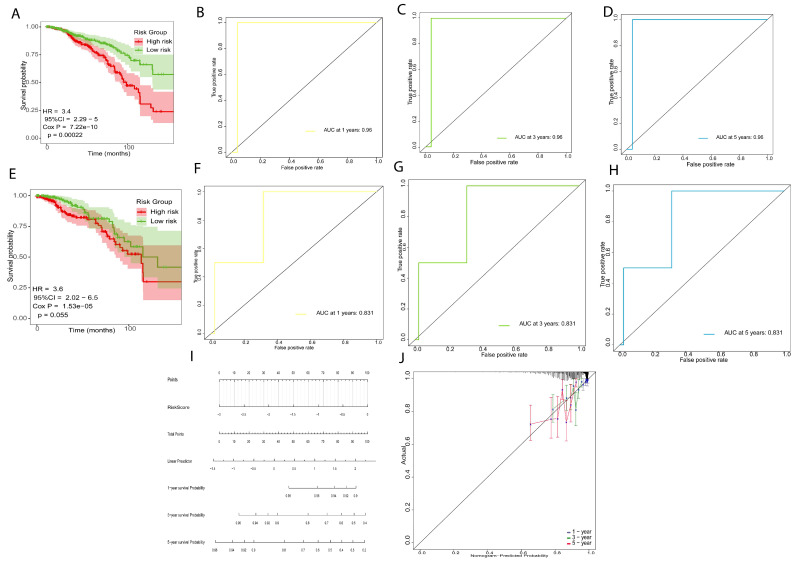
Verification of the prognostic risk signature model. (**A**–**D**) Survival analysis curve and ROC of training sets. (**E**–**H**) Survival analysis curve and ROC of validation sets. (**I**) Nomogram of prognostic risk signature model. (**J**) Nomogram calibration curves to predict 1-, 3-, and 5-year survival.

In the training set, 796 samples (n = 796), the patients in the low-risk group (n = 398) had significantly longer overall survival times (*p* = 0.00022, HR = 3.4, Figure 4A) and the AUC was 0.96 for 1-year, 3-year and 5-year (Figure 4B–D), indicating that the prognostic risk signature has high precision. We used the same method assigned to the validation set (n = 398). The patients in the low-risk group showed longer survival rates (*p* = 0.05, HR = 3.6, Figure 3E), and the AUC was 0.831 for 1-year, 3-year, and 5-year survival, which indicated that the prognostic risk model has predictive power (Figure 4F–H). Finally, we established a nomogram using the prognostic risk signature (Figure 4I). The calibration curves for 1-year, 3-year, and 5-year survival indicate a high degree of overlap between the actual survival and survival rate predicated by the nomogram (Figure 4J), suggesting that the nomogram has a significant predictive value.

### 2.4. Immune Prediction and Clinical Application of Prognostic Risk Signature

**Figure 5:**
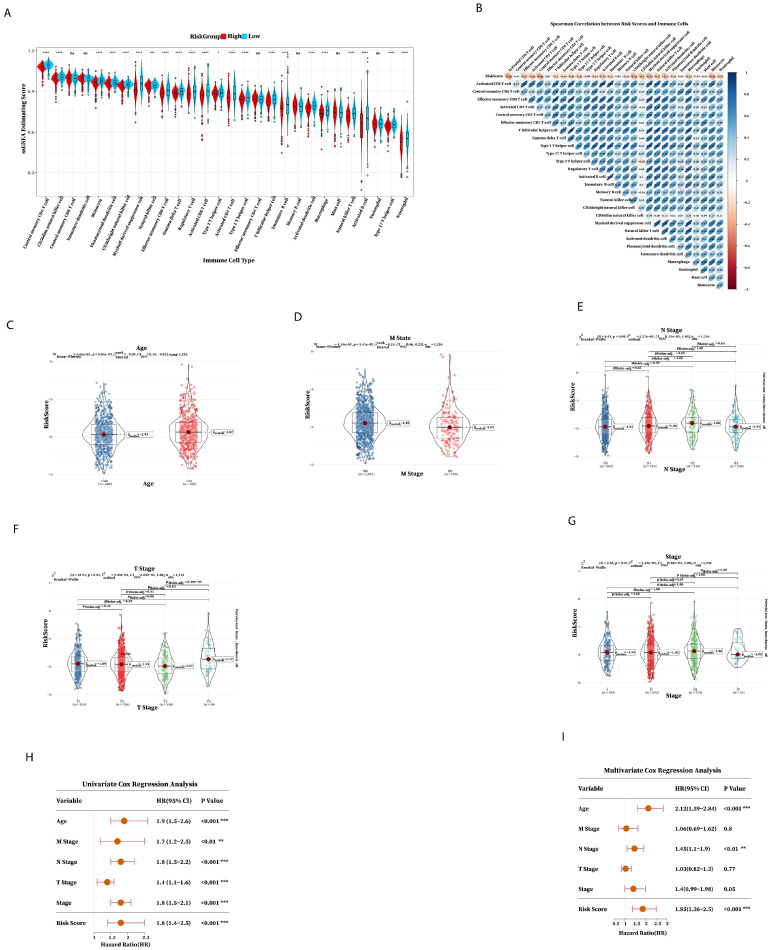
Immune predictive performance and clinical predictive power of the prognostic risk signature. (**A**) The abundance of 28 immune cells in high- and low-risk groups after grouping the risk score according to the median. (**B**) Spearman correlation analysis between the abundance of 28 cells and risk score. (**C**–**G**) Age, M state, N stage, T stage, and stage distribution of the high-risk and low-risk groups of patients. (**H**) Univariate regression analysis and (**I**) multivariate regression analysis of TCGA cohorts (* *p* < 0.05; ** *p* < 0.01; *** *p* < 0.001; **** *p* < 0.0001).

The GSA result found that the content of most immune cells in the low-risk group was higher than that in the high-risk group, indicating that there were fewer immune cells in the tumor microenvironment of the high-risk group. Also, the immune prognosis of the high-risk group was worse (Figure 5A,B); the effect of immunotherapy worsens as the risk score increases and the immune composition decreases. The above results showed that the prognostic risk signature was involved in regulating the immune microenvironment and can be used as an indicator to predict the efficacy of immunotherapy. Then, we investigated the relationship between risk score and clinical characteristics; there were significant statistics among Age, M state, and T stage (Figure 5C–G), which indicated that risk score was related to M state, T stage, and Age. Next, we explored the clinical application of seven prognostic risk signatures in predicting patient outcomes using Cox regression analysis univariate, Cox regression analysis, and multivariate Cox regression analysis to check whether the prognostic risk signature can be independently prognostic. Univariate Cox regression analysis demonstrated that the risk score was significantly associated with prognostic factors (Figure 5H), and multivariate Cox regression analysis showed that risk score was an independent factor for BRCA patients (Figure 5I). These results confirmed that the seven prognostic risk signature has excellent prognostic efficiency.

### 2.5. Functional Enrichment Analysis of Prognostic Risk Genes Signature

**Figure 6:**
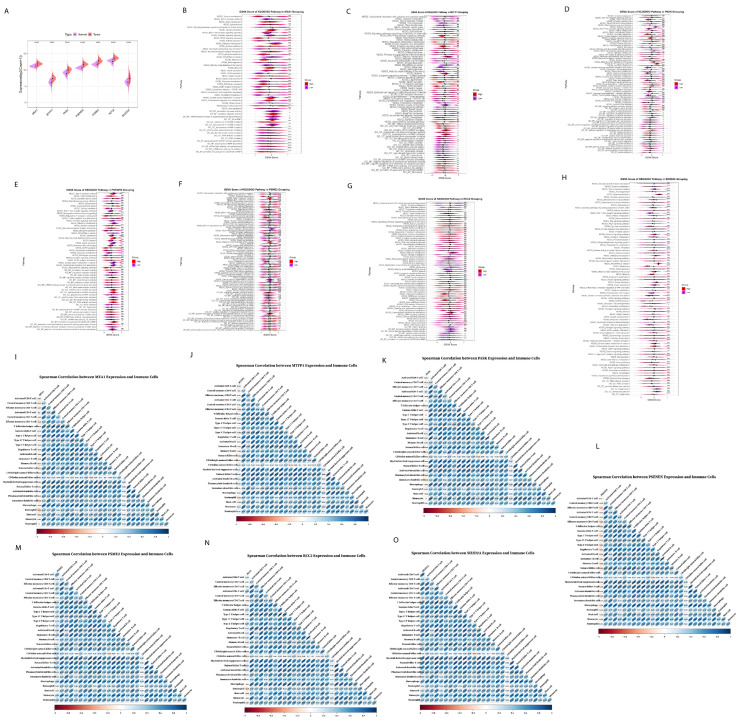
Expression analysis and Spearman correlation analysis between the abundance of 28 cells and prognostic genes, as well as functional analysis of seven prognostic genes in TCGA cohorts. (**A**) Expression level of seven prognostic genes in the TCGA cohorts. GSVA after grouping (**B**) MEAI, (**C**) *MTFP1*, (**D**) *PASK*, (**E**) *PSENEN*, (**F**) *PSME2*, (**G**) *RCC2*, and (**H**) *SH2D2A* into high and low levels. (**I**–**O**) Spearman correlation analysis between the abundance of 28 cells and seven prognostic genes. (* *p* < 0.05; ** *p* < 0.01; *** *p* < 0.001; **** *p* < 0.0001).

We further explored the prognostic risk genes expression and pathways alteration of the seven prognostic genes expressed at a low and high level in tumor tissues using TCGA BRCA cohort (Figure 6A). Firstly, for *MEA1*, it was upregulated in tumor tissue, and the results of various pathways including gastric cancer, breast cancer, basal cell carcinoma, and signaling pathways regulating the pluripotency of stem cells (Figure 6B), and *MEA1* was negatively correlated with the abundance of most immune cells (Figure 6I), indicating that *MEA1* was involved in tumor progression. Similarly, MTFP1 was expressed at a low level in tumor tissue. The results of GSVA showed that *MTFP1* upregulated IL-17 signaling pathways, calcium signaling pathways, breast cancer, negative regulation of epithelial cell apoptotic process, negative regulation of endothelial cell apoptotic process, fibrinolysis and signaling pathways regulating pluripotency of stem cells (Figure 6C); *MTFP1* showed negative correlation to most immune cells (Figure 6J), indicating that MTFP1 promotes tumor growth and progression. As for *PASK*, it showed a high expression level in tumor tissue and showed a negative correlation with the abundance of immune cells (Figure 6K); it was found that *PASK* downregulated defense response and antimicrobial humoral response, with downregulation in defense response to a Gram-positive bacterium (Figure 6D), indicating that high expression of *PASK* in tumor tissue was not suppressed by immune cells. Furthermore, *PSENEN* showed increased expression in tumor tissue and showed a negative correlation with the abundance of most immune cells using Spearman correlation analysis (Figure 6L), and central carbon metabolism in cancer and carbohydrate digestion and absorption were increased significantly (Figure 6E), which also proved the role of *PSENEN* in promoting cancer progression. After the overexpression of *PSME2*, it was found that it upregulated the IL-17 signaling pathways, chemokines signaling pathways, Corona disease COVID-19, and transcriptional mis-regulation in cancer (Figure 6F), while Spearman correlation analysis showed that they were positively correlated with the abundance of immune cells (Figure 6M), suggesting that it plays a role in various immune and inflammation responses. *RCC2* and *SHD2A2* both were highly expressed in tumor tissue: *RCC2* showed positive as well as negative correlation with the abundance of most immune cells (Figure 6N); it upregulated the tryptophan metabolism, cholesterol metabolism, and signaling pathways regulating pluripotency of stem cells (Figure 6G), implying that *RCC2* promotes cancer cell growth and differentiation. After the high expression of *SH2DA2*, it was found that it upregulates the JAK-STAT signaling pathway, PI3K-Akt signaling, chemokine signaling pathways, IL17 signaling pathways, regulation of Regulatory T cells differentiation, production of interleukin-2 production, and regulation of chronic inflammatory response (Figure 6H), and showed positive correlation with abundance of immune cells (Figure 6O), suggesting that *SH2D2A* might affect immune function.

### 2.6. Immunotherapy Outcome Prediction by Prognostic Risk Signature

**Figure 7:**
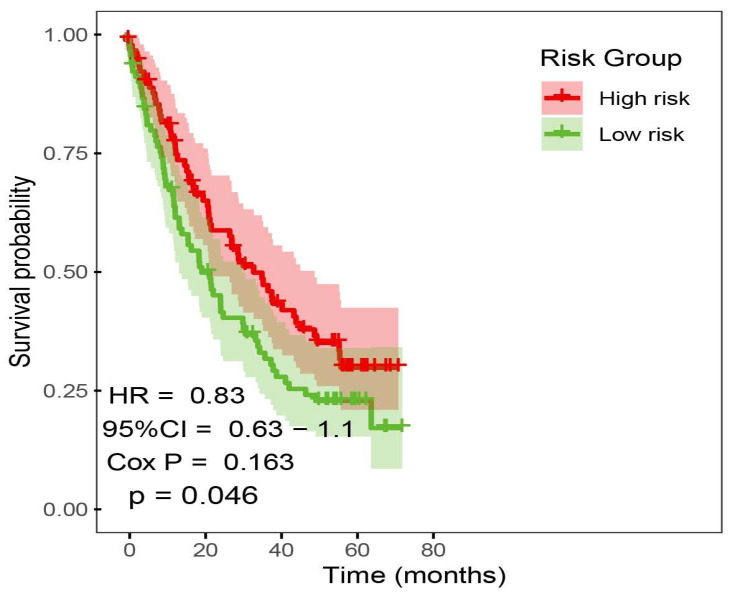
Prediction of ICI outcome using prognostic risk model. Kaplan–Meier curves comparing OS between high-risk and low-risk patients after ICI treatments.

We collected bulk RNA-seq data and clinical information from 4 ICI cohorts to investigate the predictive value of prognostic risk signature. Pre-treatment samples of 4 ICI cohorts were curated and analyzed. Patients received anti-PD(L)-1 and anti-CTLA-4. To evaluate whether the prognostic risk signature model can predict overall survival, we merged the 4 ICI cohorts into a large cohort consist on 381 samples (n = 381) and randomly selected 149 ICI-treated patient samples into high and low-risk subgroups; the Kaplan–Meier analysis of OS is shown in (Figure 7). High-risk group achieved a significantly longer survival (*p* value < 0.05).

### 2.7. Molecular Docking of Prognostic Risk Signature Genes

**Figure 8:**
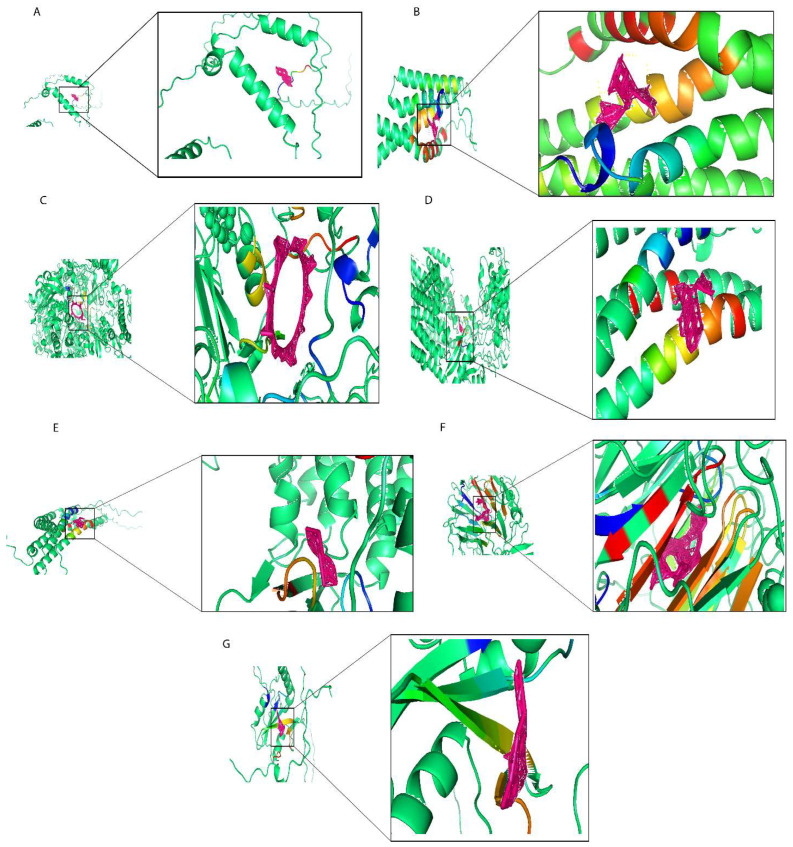
Molecular docking results of proteins encoded by prognostic risk genes with small molecular compounds. (**A**) The docking results of *MEAI* with Ethinyl Estradiol. (**B**) The docking results of *MTFP1* with epigallocatechin gallate. (**C**) The docking results of *PASK* with Cyclosporine. (**D**) The docking results of *PSENEN* Gentamicin. (**E**) The docking results of *PSME2* with Doxorubicin. (**F**) The docking results of *RCC2* with Ivermectin. (**G**) The docking results of *SH2D2A* with Dronabinol.

In this work, we used the screening of the CTD database, as well as Autodock molecular docking and drug toxicology studies to identify potential drugs targeting prognostic risk signature genes. We found that Ethinyl Estradiol binds with *MEA1* with optimal docking binding energy of −2 (kcal/mol) and downregulated *MEA1* mRNA expression (Figure 8A). Estradiol is often used in treating estrogen depletion in women. The results of molecular docking analysis indicated that epigallocatechin gallate tightly binds to *MTFP1* with a docking energy of −9.01 (kcal/mol) (Figure 8B) and downregulated the mRNA expression of *MTFP1*. Epigallocatechin gallate is a polyphenolic compound found in green tea used against neurological diseases, including Alzheimer’s disease, multiple sclerosis, and Parkinson’s disease, and it is also effective against anticancer activities. Cyclosporine actively binds with PASK with an optimal docking energy of −15.399 kcal/mol) (Figure 8C) and downregulated the mRNA expression of *PASK*. Cyclosporine is a first-line immunosuppressive drug to prevent rejection in transplantation and is also used against different cancer activities. Gentamicin downregulated the mRNA expression of *PSENEN* and exhibited docking binding energy of −6.54 (kcal/mol) (Figure 8D). Gentamicin is an aminoglycoside antibiotic used in the treatment of infectious diseases and also significantly impacted cancer treatment. Doxorubicin exhibited a high level of docking energy of up to −11.69 (kcal/mol) and downregulated the mRNA expression of *PSME2* (Figure 8E). Doxorubicin is a chemotherapy drug that slows cancer cell growth and prevents its repaid division. Ivermectin, with an optimal binding energy of −21.05 (kcal/mol), tightly binds with *RCC2* and downregulates its mRNA expression (Figure 8F). Ivermectin is a dihydro derivate of avermectin, which is more efficient against several kinds of parasitic diseases including onchocerciasis and lymphatic filariasis, and also prevents tumor cell growth and metastasis. Finally, in screening for small molecule compounds that downregulated *SH2D2A* mRNA expression (Figure 8G), Dronabinol exhibited molecular docking energy of −6.44 (kcal/mol). Dronabinol is a synthetic form of THC (Δ-9-tetrahydrocannabinol), also indicated in adults for the treatment of anorexia-associated weight loss in patients with HIV/AIDS. In summary, we have selected seven small molecular compounds suggested to improve the poor prognosis caused by the prognostic risk signature of seven genes, providing new insights into targeted breast cancer therapy.

### 2.8. Prediction of Synergistic Drug Combination by Deep Learning

We trained using data from previous studies, which included 38 drugs and 39 cell lines. According to 6:2:2, the training data is divided into training, verification, and test sets. We optimized drug coding methods and deep learning models from previous studies to be able to predict synergistic values for drug combinations used outside of the training data. Building a deep learning model with TensorFlow, we expected the synergistic effect between the two drugs in the breast cancer cell lines, and the synergistic value was more significant than 0, indicating a synergistic effect; the top twenty synergistic effects are shown in Table 1.

### 2.9. Elucidate the Expression Levels of mRNA and Protein of Prognostic Risk Signature Genes

**Figure 9:**
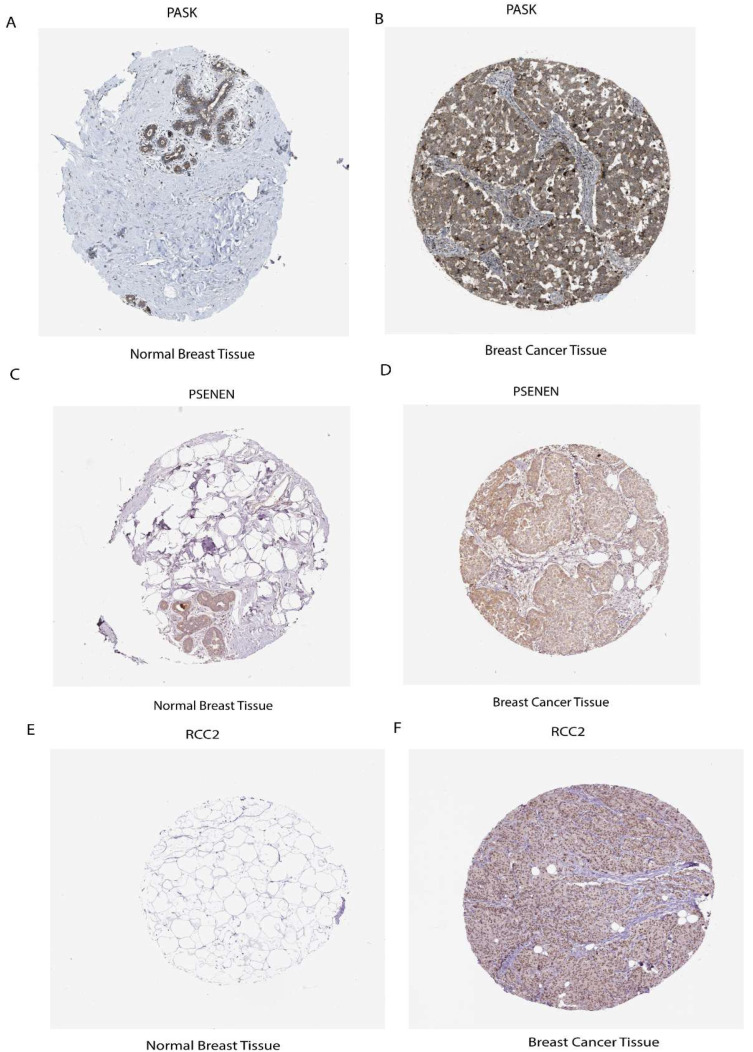
Expression level of mRNA and protein of prognostic risk signature. (**A**) *PASK* protein expression level in normal breast tissue (**B**) and breast cancer tissue. (**C**) *PSENEN* expression in normal breast tissue (**D**) and breast cancer tissue. (**E**) *RCC2* expression in normal breast tissue and (**F**) in breast cancer tissue.

To explore the clinical significance of the seven prognostic risk model-related genes, used clinical specimens from the HPA (Human Protein Atlas) database; HPA analysis showed that the protein levels *PASK*, *PSENEN*, and *RCC2* were shown in Breast cancer tissues compared to normal Breast tissue and *MTFP1*, *PSME2, MEA1,* and *SH2D2A* were not found in database. It was discovered that prognostic risk model-related three genes, including *PASK*, *PSENEN*, and *RCC2*, were upregulated in cancer tissue compared to normal Breast tissue, as shown in (Figure 9A–F).

## 3. Discussion

The rapid development of scRNA-seq in cancer and the intervention of numerous analytical methods have advanced the understanding of heterogeneous tumors, including breast cancer. Recent studies have demonstrated that the tumor immune microenvironment (TIME) has critical roles in immunotherapy [18]; further investigations of TIME are required to improve immunotherapy. Increasing attention has been focused on the powerful immune effects of Regulatory T cells (Tregs) in the tumor microenvironment (TME) [19]. Several research have reported that the infiltration of Tregs into various tumor tissues promotes cancer progression and supports cancer immune evasion by limiting antitumor immunity [18]. Immune checkpoint inhibitors (ICIs) have provided an epochal shift in the treatment of cancer [20], and most immune checkpoint molecules are expressed in Tregs [21,22]. Still, the effects of ICIs on Tregs and their contributions to treatment responses remain unclear. The involvement of Tregs is a promising anticancer strategy and an effective Treg-targeted therapy. Herein, we investigated TIME in breast cancer by focusing on the mechanism of Tregs and its association with stemness and inverse correlation with ICI outcomes.

This work comprehensively analyzed the TIME in breast cancer by scRNA-seq datasets. After quality control and dimensionality reduction clustering, 25 clusters were identified and further annotated into 12 cell types, with the subgroup clustering of T cells into eight cells and selected Tregs for further study. Further mining the high heterogeneity of Tregs, we identified three distinct differentiation states of Tregs through developmental trajectory analysis. We functionally characterize signatures of differentiation and perform GSEA analysis. We found that this differentiation pattern is intrinsically linked to intra-tumoral immune and metabolic biology, and Treg differentiation-related genes (TRDGs) showed a 97% high mutation rate in different differentiation states, indicating that TDRGs play a critical role in the development and occurrence of tumors. Based on our findings, we established a prognostic risk signature consisting of seven TDRGs (*MEA1, MTFP1, PASK, PSENEN, PSME2, RCC2,* and *SH2D2A*), which upregulates JAK-STAT signaling pathway, PI3K-akt signaling, chemokine signaling pathways, IL17 signaling pathways, regulation of Regulatory T cells differentiation, production of interleukin-2 production, regulation of chronic inflammatory response, and transcriptional mis-regulation in cancer. Overall, the risk signature can effectively predict the prognosis and immunotherapy response in breast cancer patients and provide a theoretical basis for formulating individualized treatment for BC patients.

Research has shown that *MEA1* plays an essential role in late spermatogenesis, fertilization, and sperm movement [23]. Our studies found that *MEA1* negatively correlated with the abundance of most immune cells upregulated in tumor tissue and the results of various pathways, including gastric cancer, breast cancer, basal cell carcinoma, and downregulated translation repressor activity. *MTFP1* is involved in developing and occurring oral squamous cell carcinoma; its overexpression-mediated mitochondrial fragmentation and subsequent ROS production promote cancer growth [24]. Our research found that *MTFP1* exhibits low expression in tumor tissue. The results of GSVA showed that *MTFP1* showed a negative correlation to most immune cells and upregulated IL-17 signaling pathways, calcium signaling pathways, breast cancer, negative regulation of epithelial cell apoptotic process, negative regulation of endothelial cell apoptotic process, and fibrinolysis, indicating that *MTFP1* promotes tumor growth and progression. Recent studies have shown that *PASK* is a potential therapeutic target for metabolic diseases as *PASK* plays a vital role in energy homeostasis, metabolic regulation, and nutrient sensing [25,26]. In our research, we found *PASK* showed a high expression level in tumor tissue; it was found that *PASK* downregulated defense response and antimicrobial humoral response, exhibiting a positive regulation of peptide secretion, and showed a negative correlation with abundance of immune cells, indicating that high expression of *PASK* in tumor tissue was not killed by immune cells. *PSENEN* expression was closely correlated with the infiltration of immune cells in different cancer types [27] and significantly associated with TMB, MSI, and tumor stemness index [28], which was confirmed by recent studies. In our work, we found *PSENEN* showed high expression in tumor tissue and showed a negative correlation with the abundance of most immune cells when using Spearman correlation analysis, and central carbon metabolism in cancer and carbohydrate digestion and absorption was increased significantly, which showed the role of *PSENEN* in promoting cancer. A recently reported *PSME2* Proteasome activator complex subunit 2 was positively correlated with immunomodulators, tumor mutation burden (TMB) levels, tumor-infiltrating immune cells (TIICs), and immune checkpoints in breast cancer tissue [29], and it was also associated with the invasion ability of clear cell renal cell carcinoma by inhibiting BNIP3-mediated autophagy [30].

Our study found that it upregulated the IL-17 signaling pathways and chemokines signaling pathways. Spearman correlation analysis showed that they were positively correlated with an abundance of immune cells, suggesting that they play a role in various immune and inflammation responses. *RCC2*, highly expressed in tumor tissue, showed positive as well as negative correlations with the abundance of most immune cells; it upregulated the tryptophan metabolism, cholesterol metabolism, and signaling pathways regulating pluripotency of stem cells, implying that *RCC2* promotes cancer cell growth and differentiation. Previous research reports showed that the regulator of chromosome condensation 2 (*RCC2*) is essential for stabilization and transcriptional activation of Sox2 and its overexpression is associated with cell proliferation, migration, and tumor promotion in esophageal cancer [31]. Increased radio resistance and tumor progression in Glioma via activating of transcription *DNMT1* [32] play a critical role in tumorigenesis in a range of cancers including lung cancer [33], melanoma [34], gastric cancer [35], ovarian cancer [36], as confirmed by accumulating evidence. Studies have shown that the T cell-specific adopter protein (TSAd) encoded by the *SH2DA2* gene necessary for the activation of T cell [37,38] was required for c-Src activation, vascular endothelial growth factor receptor 2 (VEGFR2)/c-Rous sarcoma (c-Src) interaction, and promoted vascular permeability in tumor vessels [39,40]. In our work, we found that it upregulates chemokine signaling pathways, IL17 signaling pathways, regulation of T cell differentiation, production of interleukin-2 production, regulation of chronic inflammatory response, and showed a positive correlation with the abundance of immune cells, suggesting that *SH2D2A* might affect immune function. Understanding the function of prognostic risk signature genes and their cause of worse prognosis will help to propose a target therapeutic approach. Our stratified analysis revealed that the prognostic risk signature model achieved significantly better survival in the high-risk group compared to low-risk group after treatment with anti-PD(L)-1 and anti-CTLA-4 using bulk RNA-seq data and clinical information from 4 ICI cohorts.

In addition, the protein expression of seven prognostic risk signature model genes was verified in breast cancer using the public HPA database. *PASK*, *PSENEN*, and *RCC2* were shown in breast cancer tissues compared to normal breast tissue, which were upregulated and had a favorable link with cancer progression. Through comprehensive analysis, this study demonstrates the robust link among Tregs, stemness, and ICI outcomes.

Nowadays, for different disease treatments, the reorientation of drug function is a novel strategy. Therefore, based on this strategy, we performed targeted therapeutic drug screening of prognostic risk signature genes, which modulates the poor prognosis. Our work demonstrated that Ethinyl Estradiol binds with *MEA1* and downregulated *MEA1* mRNA expression. Estradiol is often used in treating estrogen depletion in women and is an immunomodulator in immune and inflammatory processes [41]. Epigallocatechin gallate is a polyphenolic compound found in green tea used against neurological diseases including Alzheimer’s disease, multiple sclerosis, and Parkinson’s disease [42]; it is also effective against anticancer activities [43], tightly binds to *MTFP1*, and downregulates the mRNA expression of *MTFP1*. Cyclosporine actively binds with *PASK* with high energy and downregulates the mRNA expression of *PASK*. Cyclosporine is a first-line immunosuppressive drug to prevent rejection in transplantation and is also used against different cancer activities [44,45]. Gentamicin downregulated the mRNA expression of *PSENEN*. Gentamicin is an aminoglycoside antibiotic used in the treatment of infectious diseases and also greatly impacted cancer treatment [46,47]. Doxorubicin exhibited a high level of docking energy and downregulated the mRNA expression of *PSME2*. Doxorubicin is a chemotherapy drug that slows cancer cell growth and prevents its rapid division [48]. Ivermectin tightly binds with *RCC2* and downregulates its mRNA expression. Ivermectin is a dihydro derivate of avermectin, more efficient against several parasitic diseases, including onchocerciasis and lymphatic filariasis, and prevents tumor cell growth and metastasis [49]. Finally, in screening for small molecule compounds, Dronabinol downregulated *SH2D2A* mRNA expression. Dronabinol is a synthetic form of THC (Δ-9-tetrahydrocannabinol), also indicated in adults for the treatment of anorexia-associated weight loss in patients with HIV/AIDS [50].

Recent studies have shown deep learning success in drug discovery [51]; combination therapies are more effective in multiple diseases and infections than single doses. To achieve therapeutic solid effects and reduce side effects, synergistic combinations can improve therapeutic efficacy and potency. For example, a study by Quinn et al. discovered that sabutoclax, combined with minocycline, an antibiotic, has previously displayed synergistic effects on the intrinsic apoptotic pathway against cancer. In pancreatic ductal adenocarcinoma, this combination showed selective toxicity and reduced tumor growth in vitro and in vivo [52]. Our work predicated synergistic effects between two drugs (Ethinyl Estradiol and Doxorubicin) in breast cancer cell lines through deep learning.

The novelty of this work lies in the robust link between Tregs, stemness, and ICI outcomes analyzed via scRNA-seq data. For further investigation, discussing prognostic risk signatures’ gene function, drug targeting, and synergistic effect is also a novel strategy in this work, but at the same time, there remain limitations. First, we performed an analysis of the scRNA-seq dataset. Secondly, the combined therapeutic drugs will be the subject of future work at the cellular and animal levels; our results are still in the analytical stage and need to be experimentally validated in future work. Furthermore, based on our prognostic risk signature, we hope to develop a shared network platform that helps in therapeutic prognosis and clinical diagnosis of breast cancer.

## 4. Materials and Methods

### 4.1. Data Sources Used for Analysis

The BC (Breast Cancer) scRNA-seq dataset was downloaded from the GEO Database, including GSE176078, which contains 26 samples. scRNA-seq dataset analysis was performed using the Seurat (v4.1.1) in R. To ensure high-quality data, cells were filtered based on the following quality control criteria: cells with fewer than 500 or more than 5000 detected genes (nFeature_RNA), fewer than 200 or more than 35,000 total UMI counts (nCount_RNA), and mitochondrial gene content (percent.mt) greater than or equal to 10% were excluded from downstream analysis. First, the filtered UMI count matrix was log-transformed by executing the NormalizeData() function (scale.factor = 10,000), aiming to reduce differences in total gene counts between cells. Subsequently, FindVariableFeatures() was used to identify highly variable genes (nfeatures = 2000), selecting genes with diverse expression patterns for subsequent analysis. Dimensionality reduction was performed using RunPCA(), followed by FindNeighbors() and FindClusters(resolution = 0.4) to identify cell subtypes. The first 12 principal components were used in the RunUMAP(dims = 1:12) function to visualize the cellular heterogeneity. Finally, the FindAllMarkers() function was applied to identify marker genes for each cluster, with cutoff criteria set to adjusted *p*-value < 0.05 and absolute log2 fold change > 0.5. SingleR package (v2.0.0) and CellMarker database were used for cell type annotation. Bulk RNA-seq data were obtained from the University of California Santa Cruz (UCSC) Xena database (https://gdc.xenahubs.net), GDC TCGA breast cancer (BRCA) with dataset ID TCGA-BRCA.htseq_counts.tsv, with a total of 1217 samples and converted the RNA-seq Counts to FPKM (fragments per kilobase of transcript per million mapped reads) and normalized by log2.

### 4.2. Calculation of Stemness Index (mRNAsi)

Stemness signature was identified via one-class logistic regression (OCLR) machine learning algorithm. We used the stem cell expression profiles (syn2701943) downloaded from the Progenitor Cell Biology Consortium database (https://www.synapse.org), and subsequent Spearman correlation was conducted between stemness hallmark and normalized BC expression matrix to count the stemness index (mRNAsi) of each BC patient by scaling spearman correlation coefficients to be 0–1 accordingly.

### 4.3. Different Differentiation States of Tregs

Pseudotime trajectories of Tregs were constructed using the Monocle (v2.22.0). The algorithm uses machine learning techniques to arrange cells into trajectories with branch points based on a specific set of genes as input to place the cells into trajectories with branch points. The results are cell populations in different differentiation states, functional enrichment analysis of cells in different states, and differential analysis performed between branches. The differentially expressed genes were defined as branch-dependent or state-specific genes. These Treg marker genes located in different branch states were defined as Treg differentiation-related genes (TDRGs). Then, the somatic mutation analysis of TDRGs was performed using the maftools (v2.10.05) in R.

### 4.4. Prognostic Risk Signature Construction and Validation

We used univariate Cox regression analysis on DEGs obtained from the intersection of stemness DEG and TRDGS to construct the prognostic risk model. The genes of significance were selected, and then LASSO, the least absolute shrinkage and selection operator regression analysis using the R package “glmnet,” was used to determine the meaningful genes in the uni-Cox analysis, which defines a reliable prognostic risk model. Finally, we tested the performance of the prognostic model using a nomogram and the receiver operating characteristic curve (ROC) in R to judge the predictive accuracy of the prognostic risk model. We validated the prognostic risk model’s effectiveness using the survival analysis in R.

### 4.5. Immune Infiltration Analysis

To evaluate the relationship between immune infiltration and the prognostic risk model, we used the single-sample Gene Set Enrichment Analysis (ssGSEA) algorithm in R to calculate the degree of immune infiltration of 28 types of immune cells in the TCGA cohort to observe the relationship between the prognostic risk model and immune infiltration.

### 4.6. Functional Enrichment Analysis of Prognostic Risk Model Genes

To further explore prognostic genes in the risk model, we defined the top 30% and bottom 30% of patients with prognostic gene expression as overexpression and low-expression groups in the TCGA training cohort. Then, changes in pathway activity were analyzed by gene set variation analysis (GSVA) between the high and low groups.

### 4.7. Predictive Model for ICI Response Datasets

We analyzed four ICI RNA-seq cohorts including Hugo 2016 SKCM (n = 28) [53], Braun 2020 RCC (n = 181) [54], Liu 2019 SKCM (n = 121) [55], and Riaz 2017 SKCM (n = 51) [56], combining them to form a large cohort (n = 381). We applied ComBat method to remove the batch effect of different ICI RNA-seq cohorts and randomly selected 149 samples for analysis. The TCGA cohort (n = 1217) was randomly split into two datasets: training dataset (n = 796) was used for the prognostic risk model establishment, and (n = 421) was used as the validation set of the prognostic risk model.

### 4.8. Drugs Screening and Docking

We screened seven prognostic genes for targeted drugs based on functional studies of seven prognostic genes of the risk model. We used Autodock (Linux, v4.2) for molecular docking to study small molecule compounds interacting with prognostic genes. Firstly, we used the CTD database (https://ctdbase.org/) to download the catalog of small molecules that interacted with prognostic genes [57] and then used the PubChem database accessed on 20 December 2023 (https://pubchem.ncbi.nlm.nih.gov/) to download the small molecule structures from [58]. Next, the Uniport database (https://www.uniprot.org) was used to download the biological macromolecular structures translated by the prognostic genes [59]. Finally, the small molecule with substantial interaction with the biological macromolecules is determined by the lowest binding energy and is carried out according to the standard docking process. Moreover, PyMol (v2.6, Open-Source) visualizes the results.

### 4.9. Anticancer Drug Combination Prediction with Deep Learning

We trained using data from 22,737 (compound, compound, cell line, synergy value) quartets processed in a previous study [60], which included 38 drugs and 39 cell lines. The training data were divided into a training set, validation set, and test set according to 6:2:2. We optimized the previously studied drug coding approach and deep learning model to predict the synergy values for drug combinations used outside the training data. Firstly, by transforming the SMILES structures of 38 drugs into ECFP molecular fingerprints by RDKIT, each drug was encoded by 2048 features with radius = 6. The features of cell lines were encoded by gene expression profiling, and after normalization and filtering, each cell line consisted of 3984 features. Then, we construct the deep learning model by TensorFlow; the model consists of 5 layers of a deep network, act_func = tf.nn.relu, dropout = 0.5, input dropout = 0.2, eta = 0.00001.

### 4.10. Detection of Prognostic Gene Expression

The expression levels of seven prognostic genes were compared in breast tumor tissues and normal tissues using clinical samples from the Human Protein Atlas (HPA) database (https://www.proteinatlas.org/), using the “HPAanalyze” R package to retrieve details of the seven prognostic genes from HPA, while hpaXmlGet function was used to download the corresponding XML file for the desired genes [1] and hpaXmlTissueExpr function was used to extract the entire record of every staining available for both antibodies, including clinical data, original images, and staining quantifications.

### 4.11. Statistical Analysis

R version 4.1.1 was used for all statistical analyses. The Cox regression analysis was applied to calculate the connection between prognostic gene expression and survival outcomes. The log-rank test was used to calibrate the difference in the survival analysis, with *p*  <  0.05 indicating statistically significant difference, and Spearman Rank Correlation analysis was used to calculate correlations. Python version 3.7 was used for anticancer Drug Combination prediction, and TensorFlow constructed a deep learning model.

## 5. Conclusions

In this work, we provide solid evidence that Tregs and cancer stemness were associated with immunotherapy resistance; we analyzed the breast cancer scRNA-seq data, explored TIME, and characterized three differentiation states and TDRGs of Tregs. We established that the prognostic risk signature includes seven genes from the intersection of TDRGs and cancer stemness-related DEGs. Our prognostic risk signature could be used to predict ICI outcomes and immunotherapeutic efficacy. Lastly, some potential therapeutic drugs and Combination drugs were screened for prognostic risk signature genes, providing a new approach for target therapy.

## Figures and Tables

**Table 1:** Top twenty synergistic effects between two drugs in breast cancer cell lines.

Cell Line	Tissue	Synergy	Drug1	Drug2
T47D	BREAST	18.295	Ethinyl Estradiol	Doxorubicin
T47D	BREAST	17.79508	Gentamicin	Doxorubicin
T47D	BREAST	16.03166	Dronabinol	Doxorubicin
T47D	BREAST	14.89733	Cyclosporine	Doxorubicin
T47D	BREAST	14.25005	Epigallocatechin gallate	Doxorubicin
T47D	BREAST	12.93522	Ivermectin	Doxorubicin
T47D	BREAST	9.996137	Epigallocatechin gallate	Gentamicin
T47D	BREAST	9.803739	Gentamicin	Ethinyl Estradiol
T47D	BREAST	9.124499	Gentamicin	Dronabinol
OCUBM	BREAST	7.538448	Ivermectin	Ethinyl Estradiol
OCUBM	BREAST	7.024859	Ethinyl Estradiol	Dronabinol
T47D	BREAST	6.901407	Gentamicin	Ivermectin
OCUBM	BREAST	6.568412	Gentamicin	Ethinyl Estradiol
OCUBM	BREAST	6.47709	Ethinyl Estradiol	Cyclosporine
T47D	BREAST	5.857921	Epigallocatechin gallate	Ethinyl Estradiol
OCUBM	BREAST	4.984258	Gentamicin	Ivermectin
KPL1	BREAST	4.943874	Ethinyl Estradiol	Cyclosporine
ZR751	BREAST	4.943094	Ethinyl Estradiol	Cyclosporine
KPL1	BREAST	4.416937	Ivermectin	Ethinyl Estradiol
ZR751	BREAST	4.41583	Ivermectin	Ethinyl Estradiol

## Data Availability

All data used in this work are publicly available as described in the methodology section. The web links for publicly available datasets are described in the paper and the code used in this study is available upon request by contacting the corresponding author.

## Supplementary Materials

The following supporting information can be downloaded at: https://www.mdpi.com/article/10.3390/ijms26146995/s1.
