# Supplementary material for: Machine Learning-Based Prognostic Signature in Breast Cancer: Regulatory T Cells, Stemness, and Deep Learning for Synergistic Drug Discovery
Source: Int J Mol Sci. 2025 Jul 21;26(14):6995. doi: 10.3390/ijms26146995 (PMC12295015; doi:10.3390/ijms26146995)
Supplement: Supplementary file 1 [file ijms-26-06995-s001.zip › ijms-3738059-supplementary.pdf]

A

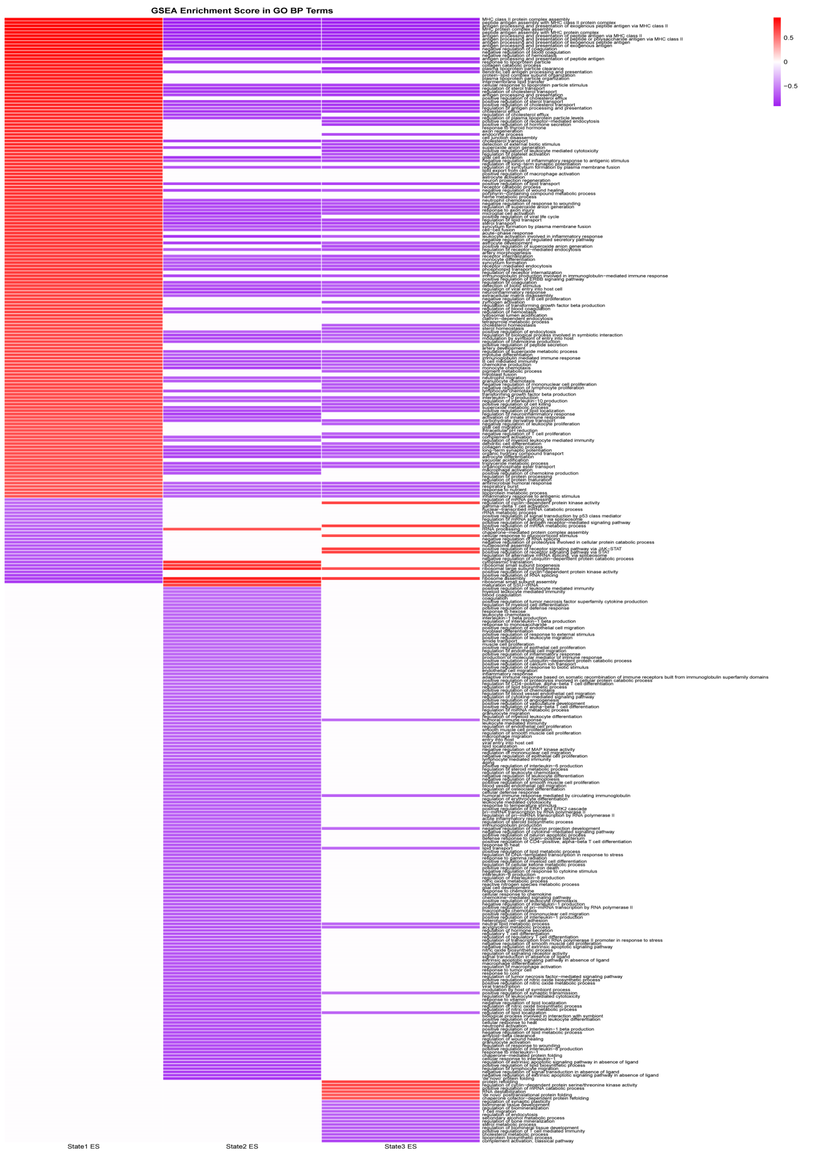

B

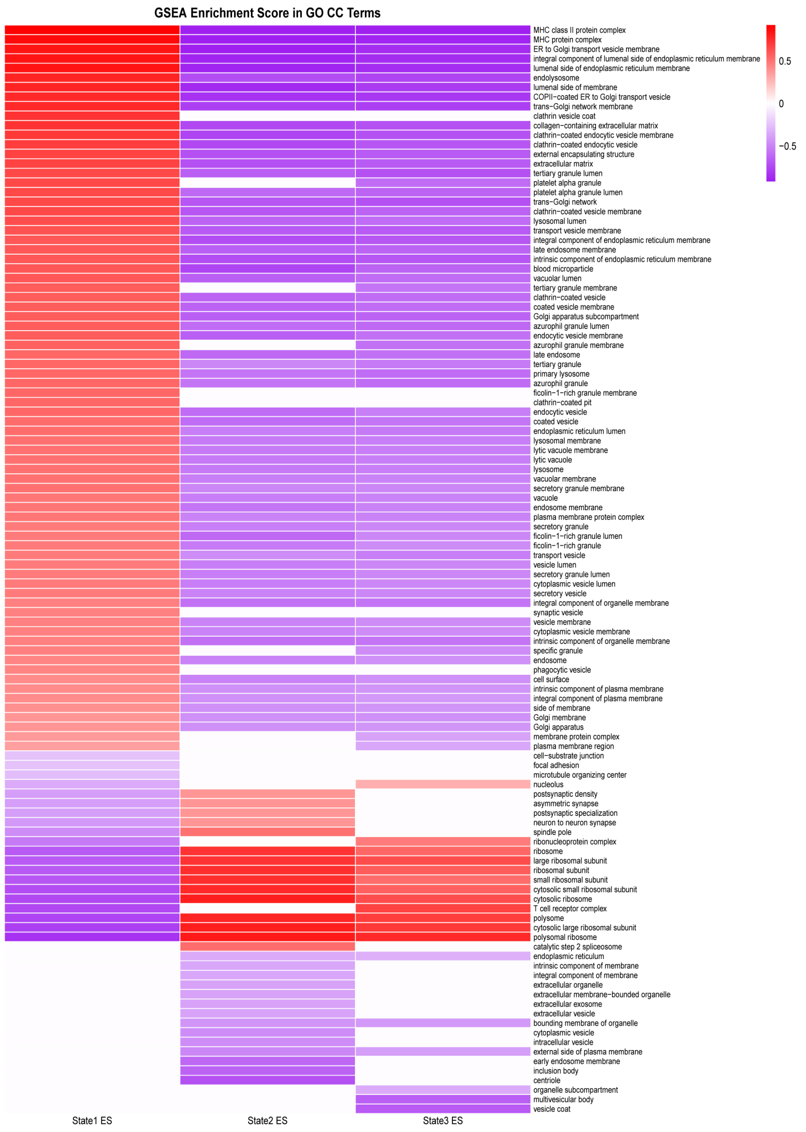


C

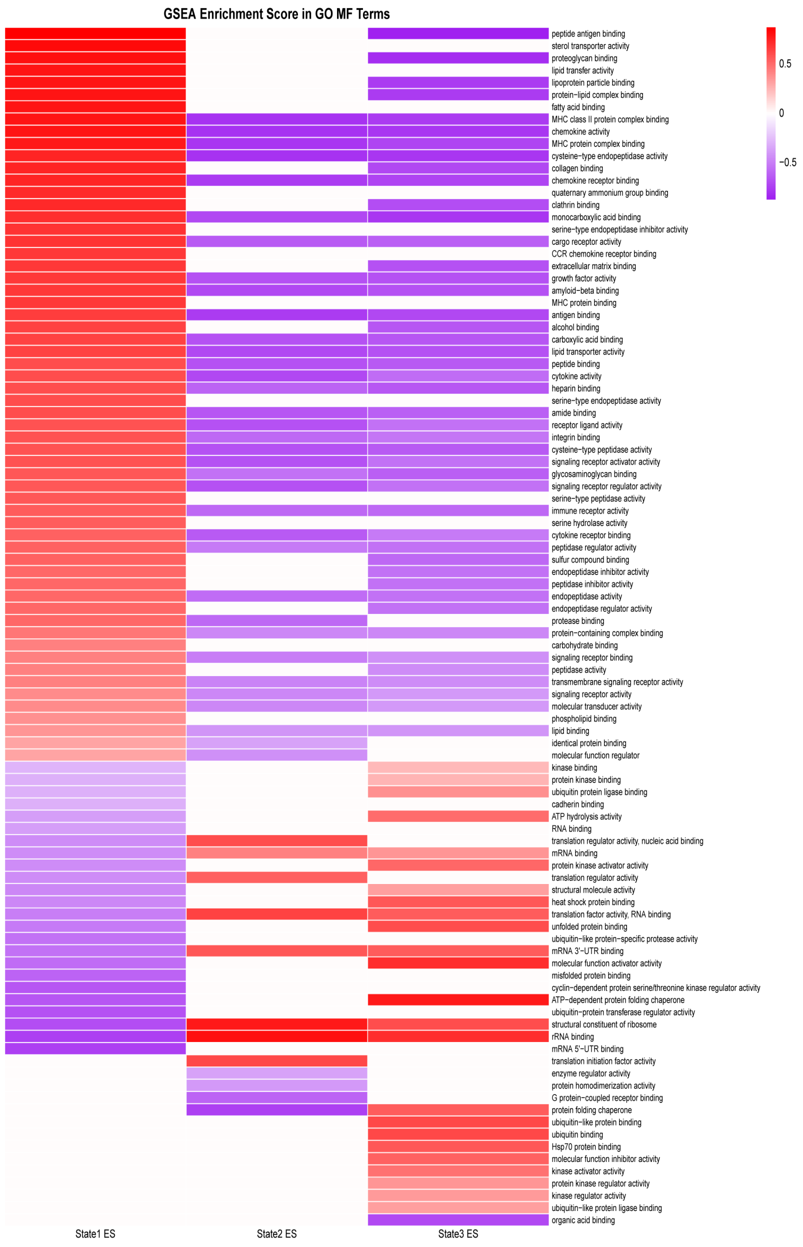


Supplementary Figure S1. GSEA analysis performed on three states of Tregs. (A) GSEA enrichment score in GO BP(B), GSEA enrichment score in GO CC. (C) GSEA enrichment score in GO MF.
